# Identifying Baseline Predictors of Selective Laser Trabeculoplasty Effectiveness: An Alternative Mathematical Approach

**DOI:** 10.7759/cureus.54116

**Published:** 2024-02-13

**Authors:** Wichapol Dendumrongsup

**Affiliations:** 1 General Practice, Faculty of Medicine, Chulalongkorn University and King Chulalongkorn Memorial Hospital, Thai Red Cross Society, Bangkok, Thailand

**Keywords:** prostaglandin analogue, trabecular outflow facility, mathematical analysis, intraocular pressure, selective laser trabeculoplasty

## Abstract

Background: Selective laser trabeculoplasty (SLT) emerges as a first-line treatment for newly diagnosed open-angle glaucoma and ocular hypertension. However, the interindividual response to SLT considerably varied. Large-scale clinical investigations concerning predictive factors for SLT effectiveness are limited. This study aimed to identify baseline predictors of the percentage intraocular pressure (IOP)-lowering effectiveness of SLT using an alternative mathematical approach.

Methods: Mathematical equations of IOP under the steady state of aqueous humour flow were formulated. The conclusive equation integrates physiological variables, including trabecular outflow facility, uveoscleral outflow fraction, plasma protein concentration, albumin/globulin ratio, mean arterial pressure, episcleral venous pressure, and plasma osmolarity. The equation was employed to estimate the percentage of IOP reduction following SLT and subsequently subjected to global sensitivity analysis to determine significant predictors of the IOP-lowering effect of SLT using the Monte Carlo simulation of 8,192 samples.

Results: In the current model, a 50% improvement in the trabecular outflow facility impacted by SLT is associated with a mean percentage IOP reduction of 16.6%. Lower baseline trabecular outflow facilities were the strongest predictors, showing a correlation with greater effectiveness of SLT in terms of percentage of IOP reduction. The second most influential factor includes baseline uveoscleral outflow fraction, followed by baseline episcleral venous pressure. Specifically, lower baseline uveoscleral outflow fraction and episcleral venous pressure were found to be correlated with increased effectiveness of SLT. Baseline levels of plasma protein concentration, albumin/globulin ratio, mean arterial pressure, and plasma osmolarity have minimal impact on SLT success or failure.

Conclusion: This study identifies baseline trabecular outflow facilities as the strongest predictor of SLT effectiveness. The results suggested that pre-SLT medical treatment that augments uveoscleral outflow and/or trabecular outflow facilities could compromise the effectiveness of subsequent SLT in terms of percentage IOP reduction compared to those who never received pre-SLT medication.

## Introduction

Glaucomatous optic neuropathy is a leading cause of irreversible blindness globally. Since intraocular pressure (IOP) is the sole modifiable risk factor for the disease, IOP-lowering strategies remain the mainstay of treatment [1]. While IOP reduction has been effectively achieved by IOP-lowering medication, long-term use raises concerns about adherence issues, side effects, and the potential impact on ocular surface changes affecting future surgical outcomes [2]. Selective laser trabeculoplasty (SLT) has recently emerged as a safe and cost-effective alternative to medications. The mechanisms underlying SLT effectiveness are theorized to involve cellular changes of pigmented cells in the trabecular meshwork induced by the application of a Q-switched 532-nm neodymium (Nd):YAG laser, resulting in an increase in aqueous outflow and IOP reduction [3]. The Laser in Glaucoma and Ocular Hypertension (LiGHT) Trial showed that patients initially receiving SLT had better long-term disease control, with reduced need for incisional glaucoma and cataract surgery over six years, compared to those being subject to solely medical therapy in the first three years. Given its efficacy, SLT positions as a first-line treatment option for patients with newly diagnosed open-angle glaucoma or ocular hypertension [1,4,5], and there has been a significant rise in the number of SLTs being conducted over the past two decades.

The interindividual response to SLT varied considerably and was unpredictable. A study involving 206 patients with ocular hypertension, primary, pseudo-exfoliation, or pigmentary glaucoma has shown IOP reduction following SLT (including both 180° and 360°) to range from 21.8 to 29.0% after six months [6]. Another study on 170 eyes with ocular hypertension and open-angle glaucoma found a mean IOP reduction of only 17.6% and 18.7% from baseline at years 1 and 2 [7]. Pre-SLT IOP has been consistently shown to be a predictor of SLT success in terms of IOP reduction [7-9]. Other variables other than pre-SLT IOP remain controversial. One large retrospective study involving a total of 997 eyes from 677 patients revealed that increased pre-SLT IOP and greater angle pigment correlate positively with SLT success. Other variables, including age, total SLT power, severity of glaucoma (defined clinically), and prior treatments, were not predictive of SLT success or failure [10]. The other study reports different predictive factors, including male sex, baseline IOP, and medical treatment before SLT [7]. To address this gap, this study aimed to identify baseline predictors of the IOP-lowering effectiveness of SLT among glaucomatous patients using a mathematical approach.

## Materials and methods

The current study applied a set of equations describing the macroscopic-scale model of aqueous flow proposed by Lyubimov et al. [11] with the following assumptions. First, the molar mass inflow rate via active transport remains constant with respect to IOP. Second, the concentration difference of low-molecular-weight species between blood and aqueous humour is relatively small. Third, ciliary capillary blood pressure is proportional to mean arterial pressure, as modelled in Szopos et al. [12]. According to Lyubimov and Stein, the equation describing aqueous flow *F* may be written as:

$F = L\left((\text{cBP} - \text{IOP}) - \sigma_p \Delta\pi_p - \sigma_s \Delta\pi_s\right) = \frac{C_o (\text{IOP} - \text{EVP})}{1-k} \quad (1)$

where $C_o$ and EVP describe trabecular outflow facility and episcleral venous pressure, respectively. $L$ represents the permeability of the membrane separating blood and aqueous humour, and cBP represents blood pressure in the capillaries of the ciliary body. $\Delta\pi_p$ and $\Delta\pi_s$ indicate the blood/aqueous oncotic pressure difference and the blood/aqueous osmotic pressure difference, respectively. $\sigma_p$ and $\sigma_s$ signify reflection coefficients for proteins and low-molecular-weight species in the blood, respectively. $k$ is defined as a dimensionless parameter describing the fraction of the uveoscleral outflow relative to the total outflow rate. It is noted that the Van't Hoff equation for a dilute solution also gives $\Delta\pi_s = \rho(C_1 - C_2)$, where $\rho$ is a product of the universal gas constant and temperature (a body temperature of 310 Kelvin is applied). $C_1$ and $C_2$ represent the total molar concentration of low-molecular-weight species in the blood and aqueous humour, respectively. Lyubimov et al. described the equilibrium state of mass production and drainage rate of low-molecular-weight species as [11]:

$F(\bar{C})(1-\sigma_s) + J = FC_2 \quad (2)$

where $J$ signifies the molar mass inflow rate via active transport, being made constant (as in the assumption), and $\bar{C} = \frac{C_1 + C_2}{2}$. The left-hand side of the equation represents the mass inflow rate (including the active transport and advection processes), while the diffusion process is neglected due to its relative smallness. The right-hand side indicates the mass outflow rate.

**Figure 1:**
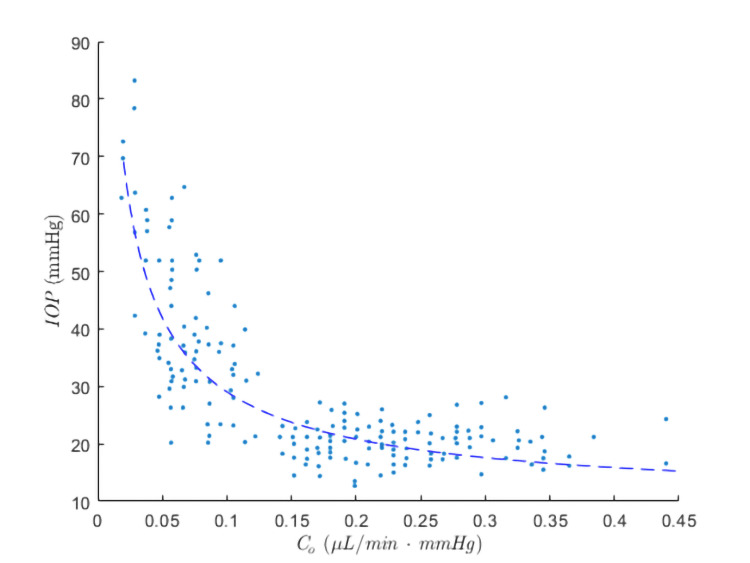
Correlation between predicted IOP using Equations (4) and (5) and clinical measurements from Grant’s study *σ_s_* = 0.055, *J* = 0.063 µmol/min, and *k* = 1/12 were selected to achieve an IOP of 20 mmHg at *C_o_* = 0.22 µL/min-mmHg and *F* = 2.4 µL/min, which represent approximate mean values in normal subjects in Grant's study (assume EVP = 10 mmHg). It is noted that the value of *σ_s_* falls within the typical range of 0.02-0.2, as indicated by Kuley et al. [10]. Other mean values used are as follows: cBP = 32.5 mmHg, *C_p_* = 7.3 g/dL, *r* = 1.5, and C_1_ = 285 mmol/L. (Partially reused figure in an unpublished manuscript under review: Dendumrongsup W, Phutinart S, Siranart N, et al. A simple mathematical model identifying determinants of intraocular pressure and latanoprost effectiveness.)

The above set of equations was analytically solved and presented in the author’s previous study, validated against clinical data from Grant [13] with good correlation (Figure 1), which can be written as

IOP = $\frac{(1 - k)F}{C_o}$ + EVP (4)

where $F = \frac{-b + \sqrt{a^2 + 4aJ}}{2a} \quad (5)$

where $a \equiv \frac{\sigma_s+1}{2\rho\sigma_s} \left(\frac{1}{L} + \frac{1-k}{C_o}\right)$ and $b \equiv \frac{\sigma_s+1}{2\rho\sigma_s}(\Delta\pi_p$ - cBP + EVP) + *C*_1_ *σ_s_*

Here, $\Delta \pi$_p_ can be written by only blood oncotic pressure (assuming no protein content in aqueous humour):

$$\Delta \pi_{p}= \frac{r}{r+1}(2.8Cp + 0.18C_{p}^{2} + 0.012C_{p}^{3}) + \frac{1}{r+1}(0.9C_{p}+0.12C_{p}^{2} + 0.004C_{p}^{3}),$$

where *C_p_* and *r* are total plasma protein concentration (g/dL) and albumin/globulin ratio, respectively [14].

According to Equation (5), it can be concluded that IOP can be written as a function, defined as IOP = *f*(*C_o_*, *k*, EVP, MAP, *C_p_*, *r*, *C*_1_). Using this notation, the percentage change in IOP from the baseline following SLT, $\% \Delta$ IOP, can be concisely written as:

$\% \Delta IOP = \left[\frac{f(\gamma C_o,k,EVP,MAP,C_p,r,C_1)}{f(C_o,k,EVP,MAP,C_p,r,C_1)} - 1\right] \times 100 \equiv G(C_o,k,EVP,MAP,C_p,r,C_1).$

For simplicity, that, again, depends on baseline parameters $C_o, k$, EVP, MAP, $C_p, r, C_1$. A parameter $\gamma$ is defined as post-SLT divided by pre-SLT trabecular outflow facility (i.e., the factor by which trabecular outflow facility increases following the procedure), which is estimated to be 50% based on previous clinical data [15,16]. It should be noted that the rationale behind increasing the trabecular outflow facility is the fact that the mechanism by which SLT lowers IOP is based on the principle of selective photothermolysis of pigmented cells of the trabecular meshwork targets without causing thermal damage to adjunctive structures [3,17]. Based on its mechanism, it may also be reasonable to assume that SLT does not affect any other parameters apart from $C_o$. To identify significant parameters with the most influences on $\% \Delta$ IOP, a global sensitivity analysis was performed on the function $G$ in Equation (6) for the OAG population. Based on previous literature [18-23], seven input parameters are assigned their distribution and associated values as follows: $C_o$, uniform, ranging 0.07-0.24 µL/min-mmHg; $k$, uniform, ranging 0.05-0.40; EVP, uniform, ranging 8-10 mmHg; MAP, normal, mean (S.D.) being 90 (10.5) mmHg; $C_p$, uniform, ranging 6.3-8.3 g/dL; $r$, uniform, ranging 1-2; $C_1$, uniform, ranging 275-295 mmol/L. It should be noted that the decision to assign MAP to follow a normal distribution was based on a large population study [24], while other parameters were assigned to be uniformly distributed to ensure they fell within a specified range.

## Results

**Figure 2:**
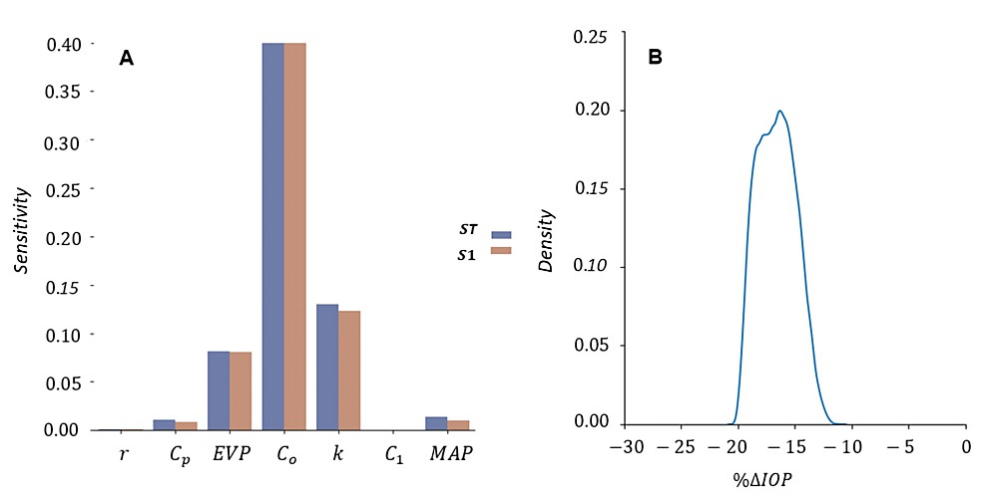
Sensitivity analysis of determinants of percentage IOP reduction following selective laser trabeculoplasty (A) and its probability density function (B). *S*_1_ and *S*_T_ denote first- and total-order Sobol indices. Parameters included in the analysis are albumin/globulin ratio, *r*; total plasma protein concentration, *C_p_*; episcleral venous pressure, EVP; trabecular outflow facility, *C_o_*; uveoscleral outflow fraction, *k*; plasma osmolarity, *C*_1_; and mean arterial pressure, MAP.

**Figure 3:**
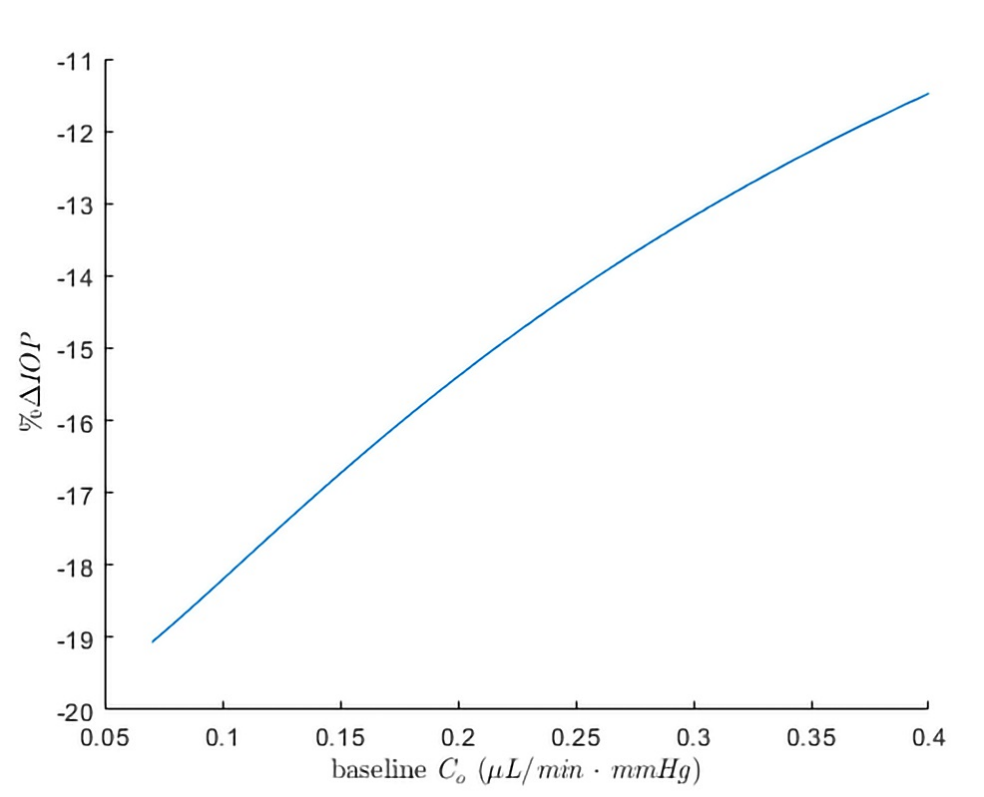
Relationships of a percentage IOP reduction following SLT with baseline trabecular outflow facility keeping other uninterested baseline parameters constant at their mean values.

Analysis of 8,192 samples from the Monte Carlo simulation revealed lower baseline trabecular outflow facilities as the strongest predictors, showing a correlation with greater effectiveness of SLT in terms of percentage of IOP reduction (Figures 2A, 3). The second most influential factor includes baseline uveoscleral outflow fraction, followed by baseline episcleral venous pressure. The probability density function of the current study’s model obtained a mean percentage IOP reduction (S.D.) of 16.6 (2.4), ranging from 11.0% to 20.4% IOP reduction (Figure 2B).

**Figure 4:**
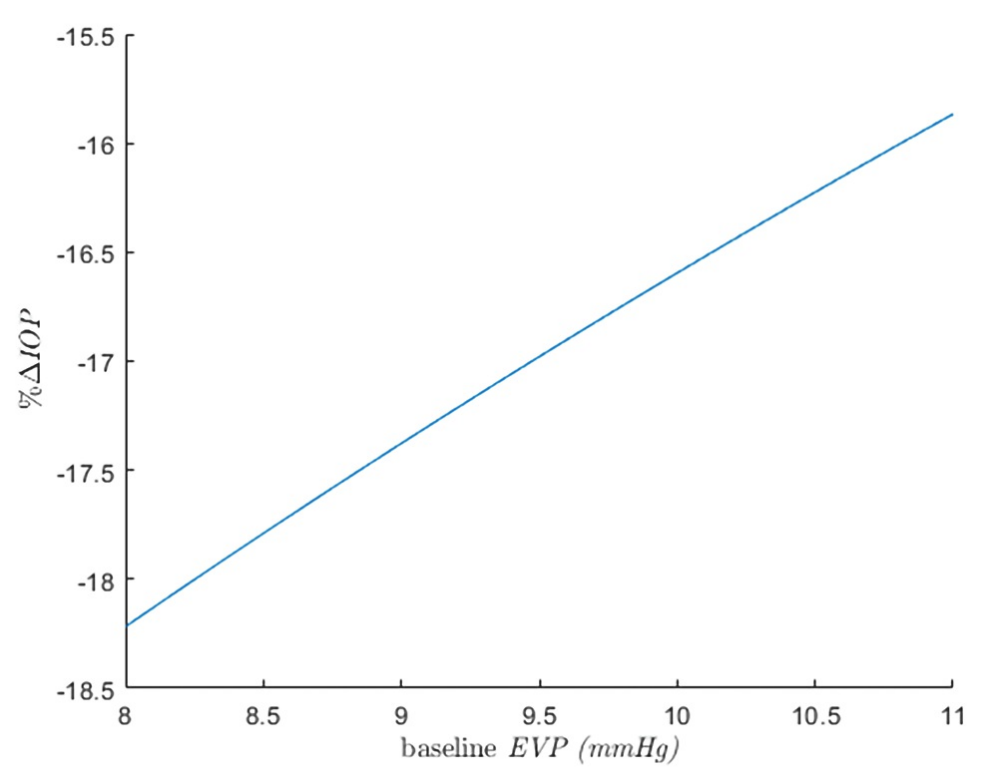
Relationships of a percentage IOP reduction following SLT with baseline episcleral venous pressure keeping other uninterested baseline parameters constant at their mean values.

**Figure 5:**
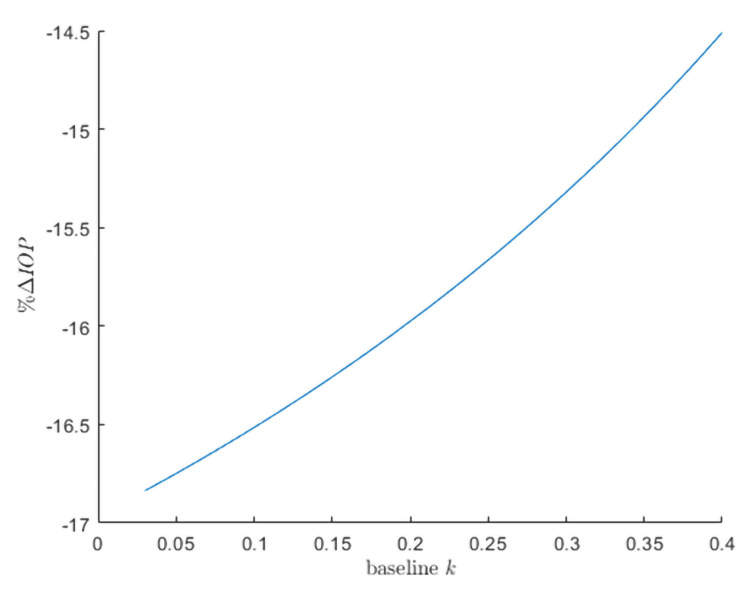
Relationships of a percentage IOP reduction following SLT with baseline uveoscleral outflow fraction keeping other uninterested baseline parameters constant at their mean values.

Specifically, lower baseline uveoscleral outflow fraction and episcleral venous pressure were found to be correlated with increased effectiveness of SLT (Figures 4-5). Baseline levels of plasma protein concentration, albumin/globulin ratio, mean arterial pressure, and plasma osmolarity have minimal impact on SLT success or failure.

## Discussion

To the best of our knowledge, this is the first study to employ a mathematical approach to investigate baseline predictors that affect the IOP-lowering effectiveness of SLT. Baseline outflow facility was found to be strongly correlated with the percentage of IOP reduction, followed by baseline uveoscleral outflow fraction and baseline episcleral venous pressure. All these factors exhibit negative correlations with the percentage of IOP reduction from SLT.

Given that lower trabecular outflow facilities are indicative of higher IOP, especially at relatively lower trabecular outflow facilities (Figure 1) [13], the results of this study confirm findings from other authors who reported that higher baseline IOP has been associated with SLT success [10,16]. Furthermore, one previous clinical study specifically found that a lower outflow facility may be predictive of a better response to SLT [16], which is in line with this study. Considering the present study, pre-SLT medical treatment that augments uveoscleral outflow and/or trabecular outflow facilities could compromise the effectiveness of subsequent SLT in terms of percentage IOP reduction compared to those who never received pre-SLT medication. These medications may involve prostaglandin analogues or Rho kinase inhibitors such as ripasudil. This is consistent with Bruen’s study, in which a reduced IOP-lowering effect of SLT in patients receiving pre-SLT treatment with prostaglandin analogue was observed after correcting for baseline IOP [25,26]. This suggests that the effectiveness of SLT may be expected to be greater in prostaglandin-naïve eyes with a lower baseline trabecular outflow facility and lower in previously prostaglandin-treated eyes with a higher baseline trabecular outflow facility.

Interestingly, it was theoretically suggested in this study that, although difficult to explain, lower baseline episcleral venous pressure could improve the efficacy of subsequent SLT. To the best of our knowledge, this observation has not been reported elsewhere. However, it should be noted that the EVP-lowering effect (approximately 10%) due to the current medication known for reducing EVP, such as netarsudil [27], may be too small to enhance subsequent SLT success with clinical significance (Figure 5).

It should be admitted that the current preliminary study has potential limitations. The primary limitation is that the current model only captures macroscopic-level dynamics of aqueous humour flow; that is, only trabecular outflow facilities are assigned to be increased due to the procedure while neglecting in-depth microscopic cellular-level dynamics, which may be involved in SLT as well. Although it should be aware that the distribution of each variable should be carefully assigned as it may affect the model outcome in general, upon the author’s trial, it is less likely to significantly change the main outcome. Moreover, the value $\gamma$, which is estimated to be 50% based on previous clinical studies, can be varied among patients. However, the reason to fix this number is that the focus of this study is to identify only baseline predictors affecting SLT success and not technique-related effectiveness. This may be the reason why the percentage reduction obtained in this study has a lower standard deviation than that observed clinically.

## Conclusions

Given that SLT has become a first-line treatment for newly diagnosed open-angle glaucoma and ocular hypertension, it is an alternative to medical treatment. It is important to better understand the predictors that affect its success. This study identifies baseline trabecular outflow facilities as the strongest predictor of SLT effectiveness. The results suggested that pre-SLT medical treatment that augments uveoscleral outflow and/or trabecular outflow facilities could compromise the effectiveness of subsequent SLT in terms of percentage IOP reduction compared to those who never received pre-SLT medication. This study should at least guide further clinical investigation on this topic in order to better select candidates for the procedure, guide clinical decisions, and inform clinicians and patients regarding the expectation of the procedural outcome.
